# Support Vector Machine and Artificial Neural Network Models for the Classification of Grapevine Varieties Using a Portable NIR Spectrophotometer

**DOI:** 10.1371/journal.pone.0143197

**Published:** 2015-11-24

**Authors:** Salvador Gutiérrez, Javier Tardaguila, Juan Fernández-Novales, María P. Diago

**Affiliations:** Instituto de las Ciencias de la Vid y del Vino, University of La Rioja, CSIC, Gobierno de La Rioja, Ctra. De Burgos Km. 6, 26007, Logroño, Spain; Universita degli Studi di Siena, ITALY

## Abstract

The identification of different grapevine varieties, currently attended using visual ampelometry, DNA analysis and very recently, by hyperspectral analysis under laboratory conditions, is an issue of great importance in the wine industry. This work presents support vector machine and artificial neural network’s modelling for grapevine varietal classification from in-field leaf spectroscopy. Modelling was attempted at two scales: site-specific and a global scale. Spectral measurements were obtained on the near-infrared (NIR) spectral range between 1600 to 2400 nm under field conditions in a non-destructive way using a portable spectrophotometer. For the site specific approach, spectra were collected from the adaxial side of 400 individual leaves of 20 grapevine (*Vitis vinifera* L.) varieties one week after veraison. For the global model, two additional sets of spectra were collected one week before harvest from two different vineyards in another vintage, each one consisting on 48 measurement from individual leaves of six varieties. Several combinations of spectra scatter correction and smoothing filtering were studied. For the training of the models, support vector machines and artificial neural networks were employed using the pre-processed spectra as input and the varieties as the classes of the models. The results from the pre-processing study showed that there was no influence whether using scatter correction or not. Also, a second-degree derivative with a window size of 5 Savitzky-Golay filtering yielded the highest outcomes. For the site-specific model, with 20 classes, the best results from the classifiers thrown an overall score of 87.25% of correctly classified samples. These results were compared under the same conditions with a model trained using partial least squares discriminant analysis, which showed a worse performance in every case. For the global model, a 6-class dataset involving samples from three different vineyards, two years and leaves monitored at post-veraison and harvest was also built up, reaching a 77.08% of correctly classified samples. The outcomes obtained demonstrate the capability of using a reliable method for fast, in-field, non-destructive grapevine varietal classification that could be very useful in viticulture and wine industry, either global or site-specific.

## Introduction

The development of a fast and automatic procedure for grapevine varietal classification would bring a new valuable way in viticulture and wine industry due to the high economical and social impact of these businesses, offering new trends on vineyard monitoring and grape quality control.

Althought classic ampelometry [1] has been widely used for grapevine varietal identification by taking morphological differences between varieties into consideration, the necessity of human expert intervention makes virtually impossible its widespread utilization. Also, wet chemistry techniques based on isoenzymes [2] or DNA analysis [3–5] have been carried out. Still, these methods are labour and time-consuming, and not able to be performed under field conditions.

Near-infrared reflectance spectroscopy (NIRs) is a non-invasive technique, highly-suited analytical method for several agricultural applications due to its rapid data acquisition time, the capability of determining more than one parameter using the same measurement, and its easy fast usage and little sample preparation.

Spectroscopy has been previously applied for fruit composition assessment [6, 7]. Also, plant varietal discrimination has been accomplished using spectroscopy in crops such as wheat [8], bayberry [9], pear [10], tomato [11] and strawberry [12], using different organs. Recent studies have explored the use of leaf spectroscopy for grapevine varietal and clone identification using hyperspectral imaging under laboratory conditions throughout destructive methods [13, 14]. Also, these works used partial least squares discriminant analysis (PLS-DA) for the training of the models.

Machine learning techniques for the development of classification models are extensively applied in countless fields. Methods such as support vector machines (SVM) or artificial neural networks (ANN) have demonstrated their high reliability in the training of non-linear regression and classification models.

SVMs have arisen as very solid machine learning methods for supervised classification issues [15]. SVMs are kernel-based algorithms that transform data into a high-dimensional space and construct an hyperplane that maximizes the distance to the nearest data point of any of the input classes. Although SVM are originally designed to train binary classifiers, an extension for multiple classes is possible by reducing the multiclass problem into several binary classification ones, using *one-versus-all* or *one-versus-one* approaches. SVM models have been used for NIR varietal classification under laboratory conditions on sesame oil [16], waxy corn seed [17] or rice seed varieties [18].

ANN are machine learning models inspired on biological neural networks present in animal brains. The first approaches of the ANN concept was exposed by [19], and afterwards resurfaced with the introduction of the *error backpropagation* concept [20, 21]. ANN are formed by units named *processing elements* (PE) having similar behaviors than a biological neuron. Different functions—as data input, output, storage or forwarding—are distributed among all the PEs. The layout of a ANN is composed of a number of layers (one-layer or multi-layer designs) and a number of PE per layer. NIR varietal classification have been carried out using ANN models in tea plants [22] or herbal medicine [23].

Most studies have developed PLS-DA based models under laboratory conditions from NIR spectroscopy obtained through destructive methods. Also, these models were constructed using only a few number of varieties as classes. The importance of fast, in-field grapevine variety discrimination using a portable device could be crucial for viticulture and wine industry. Specially in viticulture—for nurseries, appellation boards or commercial vineyards—grapevine varietal classification is a matter of great interest, e.g., discrimination of unknown vines in older vineyards, where it is usual the plantation of more than a single cultivar, or the recognition of not-allowed varieties in particular appellation regions.

The objective of this work was the classification of grapevine varieties using SVM and ANN models from in-field, portable and non-desctructive leaf NIR spectroscopy. Particularly, two approaches have been followed: the developing of a site-specific classification model for 20 grapevine varieties, comparing its performance vs a PLS-DA one; and the use of a dataset with samples from different vineyards, vintages and phenological stages for developing a global model that would cover samples from several sources.

## Materials and Methods

### Layout and experimental design

#### Site-specific model

The study was carried out on 12 August 2012 (one week after veraison) at a 1.43 ha commercial vineyard located in Vergalijo (Lat. 42^⍛^ 27’ 45.96”, Long. 1^⍛^ 48’ 13.42”, Alt. 325 m), Navarra, Spain, under permission of the owner of the vineyard plot. 20 grapevine (*Vitis vinifera* L.) varieties (Albariño, Cabernet Franc, Cabernet Sauvignon, Caladoc, Carmenere, Godello, Grenache, Malvasia, Marselan, Pedro Ximénez, Pinot Noir, Syrah, Tempranillo, Touriga Nacional, Treixadura, Verdejo, Viognier, Viura, White Grenache, White Tempranillo) were used in this study. Grapevines were trained to a vertically shoot-positioned trellis system, with North-South row orientation at 2 × 1 meters inter and intra row distances. Varieties were grafted on Richter 110 rootstock. Full irrigation was uniformly applied across the season for all varieties and these were well watered. The Relative Water Content (RWC) of leaves, measured following the method in [24], was maintained between 80% and 90% for all varieties.

#### Global model

Two additional sets of spectral measurements were acquired one week before harvest from two different vineyards, under permission of the manager and owner, located at the Rioja Regional Goverment’s Experimental Vineyard (Logroño, Spain, Lat. 42^⍛^ 26’ 4.7”, Long. 2^⍛^ 30’ 49.0”, Alt. 480 m) on 23 September 2015 and Provedo Nurseries (Viana, Spain, Lat. 42^⍛^ 27’ 52.0”, Long. 2^⍛^ 23’ 36.0”, Alt. 371 m) on 1^st^ October 2015 in order to test the behaviour and applicability of the model using samples from separated sites, vintages (samples for site-specific dataset were taken in 2012) and different stages of leaf development (August—after veraison—vs late September and October—harvest). For this model, six different varieties, grown in the three vineyard sites, were selected—Albariño, Grenache, Syrah, Tempranillo, Treixadura and Viura. At Rioja Government’s Vineyard, grapevines were trained to a vertically shoot-positioned trellis system, with Northwest-Southeast row orientation at 3 × 1.2 meters inter and intra row distances. Varieties were grafted on Richter 110 rootstock. At Provedo Nurseries, grapevines were trained to a vertically shoot-positioned trellis system, with East-West row orientation at 3 × 1 meters inter and intra row distances. Varieties were grafted on Richter 110 rootstock. Full irrigation was uniformly applied across the season for all vineyard plots and grapevines were well watered.

### Spectral measurement in the field

For the spectral acquisition, an integrated portable Near-infrared (NIR) spectral analyzer (microPHAZIR™, Thermo Fisher Scientific Inc., Waltham, MA, USA), working in reflectance mode (log1/*R*) in the range of 1600–2400 nm with an interval of 8.7 nm was used. Sensor integration time was 600 ms.

Spectral measurements were performed directly upon the adaxial surfaces of the leaves. For each leaf, five spectra were taken from different spots of the leaf blade. The mean of this five measurements was then considered as the average spectrum of the leaf. In every acquisition, the optical window of the NIR device was placed in direct contact with the surface of the leaf, making sure that the sensor window was completely covered. To avoid the contamination of the adaxial surfaces with external pollutants, vinyl gloves were used at all times when handling the leaves.

For each one of the 20 varieties for the site-specific model, 10 vines and two adult leaves per vine of the mid-upper part of the shoot (nodes 6 to 12), a total of 20 leaves per variety, were selected and labeled with its variety name in order to be measured with a portable spectrometer device. Spectra were acquired under field conditions directly on the vine in a non-destructive way. A total of 400 leaves were measured.

For each one of the six varieties for the global model, four vines and two adult leaves per vine of the mid-upper part of the shoot (nodes 6 to 12), a total of eight leaves per variety, were selected and labeled with its variety name in each of the three vineyard plots sample. Spectra were acquired under field conditions directly on the vine in a non-destructive way. A total of 144 leaves (three places, six varieties per place, four vines per variety, two leaves per vine) were measured.

### Spectral pre-processing and algorithms for modelling

The following pre-processing techniques and algorithms were used:

#### Scatter correction

Standard normal variate (SNV) followed by de-trending [25] [26] has been commonly used to remove the multiplicative interferences of scatter in the spectral signal. In SNV, average and standard deviation of all the data points are calculated individually for each spectrum. Then, the average value is substracted from the absorbance (zero-mean or centering) and the result is divided by the calculated standard deviation. De-trending subtracts from the data points a second degree least-squares fit polynomial calculated from the original data. Sometimes, no scatter correction is performed upon the raw spectra, hence two options for scatter correction were tested in this work: the application of SNV + De-trending (SNV+D) on the spectra before any other filtering, and the total omission of scatter correction (NoSNV+D).

**Fig 1:**
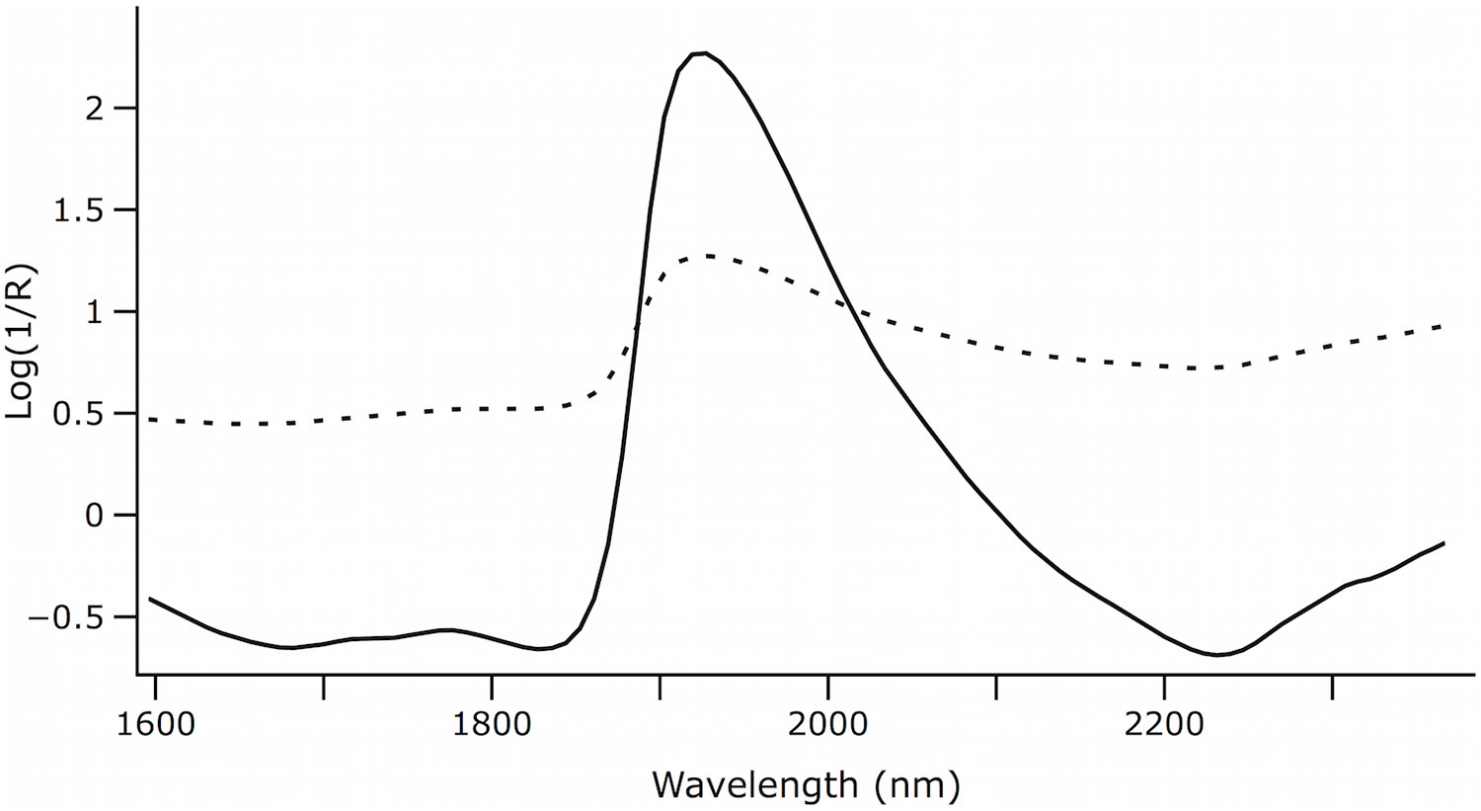
Average raw and pre-processed spectra with SNV+de-trending from the whole set of samples. Solid line: SNV+de-trending. Dashed line: Raw.

Fig 1 plots, from the 400 samples, the average raw spectrum and the result of SNV + De-trending scatter correction.

#### Smoothing filtering

A lowpass smoothing filter that makes use of local least-squares polynomial approximation was developed in [27]. This method is able to preserve the shape, height and width of waveform peaks. Savitzky-Golay filtering is usually followed by a first- or second-degree derivative. The smoothing is performed by using a moving window along the whole spectral signal. Typical values for this are in the range from 5 to 11, both included. In this work, the combination of two different derivative degrees (first-degree and second-degree) and two window sizes (5 and 11) were tested. So, four final parameter sets were defined for Savitzky-Golay filtering: first-degree derivative, window size 5 (D1W5); first-degree derivative, window size 11 (D1W11); second-degree derivative, window size 5 (D2W5); second-degree derivative, window size 11 (D2W11).

#### Classification algorithms

Two different machine learning classification algorithms (support vector machines and artificial neural networks) have been tested and compared with partial least squares discriminant analysis, widely used in spectroscopy. Sequential minimal optimization algorithm [28] working with a polynomial kernel was used in this study to train a SVM for varietal classification. Also, a multilayer perceptron was utilized as ANN classification method [29]. Partial least squares discriminant analysis (PLS-DA) has been extensively applied for classification problems, such as strawberry varieties [12], cooked ham quality [30], detection of expired vacuum-packed smoked salmon [31], Alpaca wool samples [32] or olive varietal identification [33]. PLS-DA expects to find a proper correlation of spectral variations and a set of defined classes. This is done by maximizing the covariance value between different class variables and rejecting variance within a class. In this study, PLS-DA models were trained with a maximum number of six latent variables and a uncertainty factor of 2.326.

For every one of the algorithms, the inputs were provided as the data points of the spectra (absorbance values of wavelengths 1600 nm to 2400 nm, with a step of 8.7 nm) and the classes were the identity labels of each variety.

One-way ANOVA was performed when comparing means for testing the influence of: (i) the application of a scatter correction method; (ii) the four Savitzky-Golay parameter values; (iii) the three classification algorithms and the interaction of the two previous factors. Tukey’s range test was used as mean comparison method at a significance level *p* = 0.05.

Spectra pre-processing was done using the language and software environment *R*, version 3.1.3, and the additional packages *prospectr* [34] and *pracma* [35]. *Java Language Programming*, version 1.7, along with *Weka*, version 3.6, [36] were used for machine learning algorithms executions. PLS-DA models were developed using *WinISI* software package, version 1.5 (Infrasoft International, Port Matilda, PA, USA). Finally, statistical test were performed with *InfoStat* software (Córdoba, Argentina), version 2015.

### Model training

A site-specific model for the classification of 20 varieties from the same vineyard plot was developed. In order to test the influence of the dataset population size in classifiers’ behavior, two datasets were used for training and evaluating the machine learning models: 20 varieties (N = 20 varieties and n = 400 leaves) and a randomly selected subset of five varieties (N = 5 varieties and n = 100 leaves). An execution was performed for each one of the combination of datasets, with (SNV+D) and without (NoSNV+D) scatter correction, Savitzky-Golay filtering pretreatments (D1W5, D1W11, D2W5 and D2W11), algorithms (SVM and ANN) and their parameter sets (12 sets per algorithm), making a total of 384 executions. Due to the high number of samples (leaves), a *k*-fold with *k* = 5 was selected as Cross Validation method instead of the classic number of *k* = 10.

A global dataset with six varieties—24 leaves per variety—was built up using samples from the three vineyard plots all the measurements of this work were taken (Vergalijo 2012, Logroño and Viana 2015). The total number of samples was 144 (three places, six varieties per place, eight leaves per variety). The same pre-processing algorithm and parameter set combinations were used as those employed for the site-specific model training, using again a 5-fold Cross Validation.

As previously mentioned, 12 parameter sets per machine learning algorithm have been used in order to test their influence in the classification results. These parameters are:

For SVM:
**C:** The trade-off between complexity of decision rule and frequency of error [15].
**Exponent:** The polynomial kernel exponent.
For ANN:
**Hidden layer layout:** The number of PEs present in the hidden layer of the network. *0*: no PEs (so, no hidden layer. Input PEs are directly connected to output PEs. No hidden layer layout can cast good results if the data is linearly separable). *a*: the number of PEs is the half of the sum of the number of attributes (inputs) and classes (outputs). *i*: the number of PEs is equal to the number of attributes. *o*: the number of PEs is equal to the number of classes.
**Learning rate:** It affects the speed that the minimum solution is reached by the ANN. Its value should be in the range [0, 1].
**Momentum:** It regulates the ANN capability of reaching a local minimum. The lower momentum value is set, the less likely the ANN converges to a local minimum (but more computational time will require). Its value must be in the range [0, 1].

The values used in this study for the above parameters are shown in Table 1.

**Table 1:** Parameter sets for SVM and ANN algorithms.

	**SVM**		**ANN**		
	**C**	**Exponent**	**Hidden Layer** ‡	**Learning Rate**	**Momentum**
**Parameter set 1**	3.5	1	*0*	0.3	0.1
**Parameter set 2**	0.1	1	*0*	0.3	0.9
**Parameter set 3**	1	1	*0*	0.7	0.1
**Parameter set 4**	10	1	*a*	0.3	0.1
**Parameter set 5**	3.5	2	*a*	0.3	0.9
**Parameter set 6**	0.1	2	*a*	0.7	0.1
**Parameter set 7**	1	2	*i*	0.3	0.1
**Parameter set 8**	10	2	*i*	0.3	0.9
**Parameter set 9**	3.5	3	*i*	0.7	0.1
**Parameter set 10**	0.1	3	*o*	0.3	0.1
**Parameter set 11**	1	3	*o*	0.3	0.9
**Parameter set 12**	10	3	*o*	0.7	0.1

^‡^ *0*: no processing elements (PEs) in hidden layer; *a*: PEs = (#attributes + #classes)/2; *i*: PEs = #attributes; *o*: PEs = #classes.

SVM: Support Vector Machine; ANN: Artificial Neural Network.

A source code written in *Java* was developed for the automatic running of these combinations, taking a computation time of 5 hours and 45 minutes with the following hardware specifications: Intel Core i3, 2.93 GHz processor; 12.0 GB of RAM.

## Results

The influence of scatter correction, algorithms, and smoothing filtering was statistically tested with the dataset acquired for the site-specific mode.

### Influence of scatter correction

Table 2 shows the mean comparison of the percentage of correctly classified grapevine leaves according to their variety for each algorithm when the scatter correction pre-processing was applied or omitted. For almost every case, the use of scatter correction had no statistical influence in the correctly classified percentage obtained, regardless of the algorithm. Only in the reduced dataset (N = 5), the application of SNV + De-trending performed significantly better when the SVM was used as classifier.

**Table 2:** Comparison of means of percentage of correctly classified leaves for signal scatter correction attending to the algorithm used for N = 20 and N = 5 datasets.

**Number of varieties**	**Scatter correction**	**PLS-DA**	**SVM**	**ANN**
**N = 20**	**SNV+D**	44.8	75.8	78.4
	**NoSNV+D**	47.2	75.1	77.9
	**significance**	*n.s*.	*n.s*.	*n.s*.
**N = 5**	**SNV+D**	76.5	87.9 *a*	87.1
	**NoSNV+D**	75.8	85.1 *b*	86.7
	**significance**	*n.s*.	* *	*n.s*.

*n.s*.: not significant (*p* ≥ 0.05); * *: *p* < 0.01; (Tukey’s range test at a significance level *p* = 0.05). SNV+D: Standard Normal Variate followed by De-trending; PLS-DA: Partial Least Squares Discriminant Analysis; SVM: Support Vector Machine; ANN: Artificial Neural Network.

### Influence of algorithms and Savitzky-Golay filters

Table 3 shows the comparison of means of correctly classified percentages attending to the algorithms and Savitzky-Golay pre-processing. Although there was a common and logical widespread worse response from the algorithms when the number of varieties to be classified is noticeably large, the SVM and ANN classifiers still achieved very good results for N = 20. When—for these two algorithms—the correctly classified percentages reached excellent numbers with N = 5 (values moving between 79.3% and 91.6%), this remarkable behaviour remained with a slight degradation when the number of classes was four times greater (correctly classified percentage values between 67.3% and 84.3%).

**Table 3:** Correctly classified percentages of grapevine leaves for each Savitzky-Golay filter and algorithm combination for N = 20 and N = 5 number of varieties.

**Number of varieties**	**Savitzky-Golay filter**	**PLS-DA**	**SVM**	**ANN**	**significance**
**N = 20**	**D1W5**	45.3 B	77.5 A *a*	81.2 A *b*	* * *
	**D1W11**	44.4 B	69.7 A *b*	67.3 A *c*	* * *
	**D2W5**	51.0 B	78.0 A *a*	84.3 A *a*	* * *
	**D2W11**	43.2 B	76.7 A *a*	79.8 A *b*	* * *
	**significance**	*n.s*.	* *	* * *	
**N = 5**	**D1W5**	81.5 *a*	87.8 *b*	88.0 *b*	*n.s*.
	**D1W11**	72.5 B *b*	79.3 A *c*	81.0 A *c*	*
	**D2W5**	84.5 B *a*	91.2 A *a*	91.6 A *a*	* * *
	**D2W11**	66.0 B *c*	87.8 A *b*	86.9 A *b*	* * *
	**significance**	* *	* * *	* * *	

The values shown are the varieties correctly classified percentage. Each value is, in turn, the average of the results obtained using and not using scatter correction and, for SVM and ANN, the 12 parameter sets.

PLS-DA: Partial Least Squares Discriminant Analysis; SVM: Support Vector Machine; ANN: Artificial Neural Network.

Uppercase and italic lowercase letters attend respectively to row-wise (comparison among algorithms) and column-wise (comparison among Savitzky-Golay filters) values comparison. *n.s*.: not significant (*p* ≥ 0.05); *: *p* < 0.05; * *: *p* < 0.01; * * *: *p* < 0.001.

As it can be seen, both ANN and SVM algorithms performed widely better than PLS-DA. Though this classifier obtained notable results in the reduced dataset, the increase of the number of varieties heavily degraded the output of the algorithm, barely reaching the 50% correctly classified mark (Table 3).

The results in Table 3 show that the best behaviour was generally yielded by ANN, achieving up to an average of 84.3% correctly classified instances for N = 20 and 91.6% for N = 5. Statistical tests displayed that in every case, the use of ANN or SVM casts significantly better outputs than PLS-DA, demonstrating the high suitability and surpassing response from the two machine learning classifiers vs PLS-DA. The interaction of the algorithm and the Savitzky-Golay configuration was calculated for both N = 20 and N = 5 through statistical tests. For the first dataset, the test showed that the interaction of both factor was significant at *p* < 0.05. For N = 5 dataset the interaction was significant at *p* < 0.01. If the ANOVA was performed only for SVM and ANN (ignoring the PLS-DA results), a *p*-value of 0.024 for N = 20 and 0.649 for N = 5 (not shown in the table) was obtained. Thus, while in the first case the use of ANN was significantly better (*) with regard to SVM, for N = 5 there was no significantly difference (*n.s*.) in using any algorithm.

Regarding the four different Savitzky-Golay configurations, statistical tests showed that the parameter values selection for this pre-processing filter was significantly influential in the final output, except for PLS-DA algorithm having N = 20. Also, Table 3 displays that, in every one of the cases, the Savitzky-Golay configuration with higher correctly classified percentage was a second-degree derivative with a window size of 5 (D2W5). It is likewise consistent that the second best configuration fell upon a first-derivative and window size 5 Saviztky-Golay filtering (D1W5). Using a window size of 11 returned the worst outcomes for every algorithm, so it was an avoidable choice for this study (Table 3).

### Site-specific grapevine variety classification

**Fig 2:**
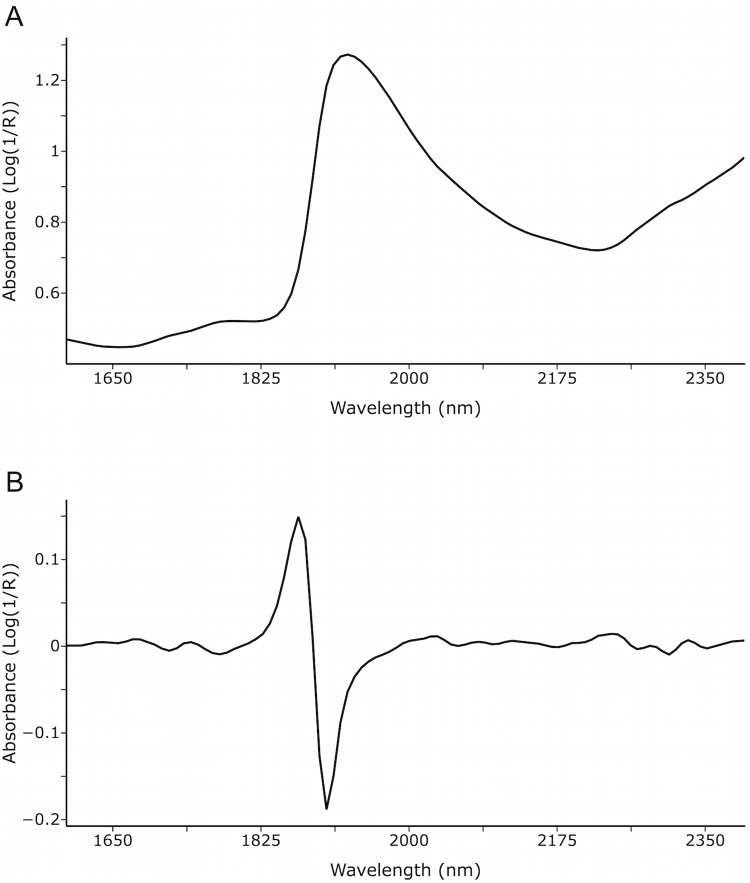
Average raw (A) and processed spectra (B) with SNV+de-trending+Savitzky-Golay filter (second-degree derivative, window size 5) from all samples.

From the 192 executions for N = 20, the best result (the one with the highest correctly classified percentage) was obtained with the following configuration: ANN, SNV+D, D2W5, parameter set 10 (Fig 2 shows, for the 400 leaf samples, the average raw and processed spectra using the pre-processing from this combination). The overall correctly classified percentage was 87.25 (349 out of 400 samples properly classified) and the confusion matrix of this configuration is shown in Table 4.

**Table 4:** Confusion matrix from the execution with the best score (ANN, SNV+D, D2W5 and parameter set 10) with an overall correctly classified value of 87.25% (20 leaves per variety).

	**Classified as**																				
	**Ve**	**M**	**V**	**A**	**T**	**G**	**WG**	**WT**	**PX**	**Vi**	**CF**	**Gr**	**CS**	**C**	**S**	**Te**	**PN**	**Ca**	**Ma**	**TN**	**%**
**Ve**	**10**	1	3	0	0	1	1	1	0	0	1	0	0	0	0	2	0	0	0	0	50
**M**	0	**14**	5	0	0	0	0	0	0	0	0	0	0	0	1	0	0	0	0	0	70
**V**	1	3	**15**	0	1	0	0	0	0	0	0	0	0	0	0	0	0	0	0	0	75
**A**	0	0	0	**19**	0	0	0	0	0	1	0	0	0	0	0	0	0	0	0	0	95
**T**	0	0	0	0	**19**	0	0	0	0	0	0	0	0	0	0	0	1	0	0	0	95
**G**	0	0	0	0	0	**18**	1	0	0	0	0	0	0	0	0	0	1	0	0	0	90
**WG**	0	0	0	0	0	0	**16**	0	0	1	0	2	0	0	0	0	0	0	0	1	80
**WT**	0	0	0	0	0	1	0	**17**	0	0	0	0	0	1	0	0	0	0	0	1	85
**PX**	0	0	1	0	0	0	0	0	**18**	1	0	0	0	0	0	0	0	0	0	0	90
**Vi**	0	0	0	0	0	0	0	0	0	**19**	0	0	0	0	1	0	0	0	0	0	95
**CF**	0	0	0	0	0	0	0	0	0	0	**20**	0	0	0	0	0	0	0	0	0	100
**Gr**	0	0	0	0	0	0	0	0	0	0	1	**19**	0	0	0	0	0	0	0	0	95
**CS**	0	0	0	0	0	0	0	0	0	0	0	0	**20**	0	0	0	0	0	0	0	100
**C**	0	0	0	1	0	0	0	0	0	0	0	0	0	**19**	0	0	0	0	0	0	95
**S**	0	0	0	0	0	0	0	0	0	0	1	0	1	0	**18**	0	0	0	0	0	90
**Te**	0	0	0	0	0	0	1	1	0	0	0	0	0	0	0	**17**	1	0	0	0	85
**PN**	0	1	0	0	0	0	0	0	0	0	0	0	0	0	0	0	**18**	0	1	0	90
**Ca**	0	0	0	0	0	0	0	0	0	0	0	1	0	0	0	0	0	**19**	0	0	95
**Ma**	0	1	0	0	0	0	0	0	0	1	0	1	0	0	1	0	2	0	**14**	0	70
**TN**	0	0	0	0	0	0	0	0	0	0	0	0	0	0	0	0	0	0	0	**20**	100

Each row represents the actual variety and in which one was classified. Bolded values (diagonal of the matrix) are the number of samples properly classified. The last column shows the correctly classified percentage for each variety.

ANN: Artificial Neural Network; SNV+D: Standard Normal Variate followed by De-trending; D2W5: Second-degree derivative and window size 5 Savitzky-Golay filter.

Ve: Verdejo; M: Malvasia; V: Viura; A: Albariño; T: Treixadura; G: Godello; WG: White Grenache; WT: White Tempranillo; PX: Pedro Ximénez; Vi: Viognier; CF: Cabernet Franc; Gr: Grenache; CS: Cabernet Sauvignon; C: Carmenere; S: Syrah; Te: Tempranillo; PN: Pinot Noir; Ca: Caladoc; Ma: Marselan; TN: Touriga Nacional.

Three varieties (Cabernet Franc, Cabernet Sauvignon and Touriga Nacional) achieved a perfect score, while six ones reached excellent results (percentage of correct classification greater than 90%): Albariño, Treixadura, Viognier, Grenache, Carmenere and Caladoc. Seven varieties obtained good results in their classification (greater or equal than 75% and less or equal than 90%): Viura, Godello, White Grenache, White Tempranillo, Pedro Ximénez, Syrah, Tempranillo and Pinot Noir. Finally, the three varieties with worst result (less than 75%) were Verdejo, Malvasia and Marselan. The classification of the Verdejo leaves resulted in a moderate output, with a 50% of correctly classified samples. From these 10 misclassified instances, three were predicted as Viura and two as Tempranillo. For Malvasia, the majority of the misclassified samples were also assigned to the Viura class.

### Global variety classification

From the 6-class global dataset model involving samples from different vineyards, vintages and phenological stages, the outcome with the highest correctly classified percentage was achieved with the following combination: ANN, NoSNV+D, D2W5, parameter set 6; reaching an overall result of 77.08% of correctly classified samples (111 out of 144). Table 5 shows the confusion matrix of this model.

**Table 5:** Confusion matrix from the global dataset execution with the best score (ANN, NoSNV+D, D2W5 and parameter set 6) with an overall correctly classified value of 77.08% (24 leaves per variety).

	**Classified as**						
	**V**	**Gr**	**T**	**Te**	**S**	**A**	**%**
**V**	**15**	1	3	0	1	4	62.5
**Gr**	0	**21**	0	2	1	0	87.5
**T**	0	0	**22**	1	0	1	91.7
**Te**	1	3	1	**17**	2	0	70.8
**S**	1	0	0	6	**16**	1	66.7
**A**	0	0	1	0	3	**20**	83.3

Each row represents the actual variety and in which one was classified. Bolded values (diagonal of the matrix) are the number of samples properly classified. The last column shows the correctly classified percentage for each variety.

ANN: Artificial Neural Network; NoSNV+D: No application of Standard Normal Variate followed by De-trending; D2W5: Second-degree derivative and window size 5 Savitzky-Golay filter.

V: Viura; Gr: Grenache; T: Treixadura; Te: Tempranillo; S: Syrah; A: Albariño.

Individually, the best classification results were obtained for Tempranillo and Grenache varieties, with a 91.7% and 87.5% correctly classified score respectively. Nonetheless, two varieties—Viura and Syrah—obtained a more modest score of correctly classified percentage, below the 70% mark in both cases.

## Discussion

The present work has shown the possibility of grapevine varietal classification using a portable NIR spectrophotometer in the field along with SVM and ANN models. 20 different grapevine varieties were classified with an overall correct classification percentage of 87.25% in a site-specific approach. Similar recent studies such as [13] or [14] also reached high percentages in varietal (93.53%) and clone (98.8%) discrimination. Nevertheless, in [13], hyperspectral imaging was conducted with a camera operating between 380 nm and 1028 nm under laboratory conditions on leaf discs. The PLS-DA models obtained in [13] were trained for the discrimination of three grapevine varieties and resulted, for every one of then, in correctly classification percentages over 92%. Although a lower overall percentage was obtained in the present work (87.25%), it is important to highlight the fact that this result was achieved from training a model with 20 classes under field conditions. Only less than five points were lost when using a classifier that had more than six times the number of classes in [13]. Additionally, attention must be drawn to the fact that the present study achieved the grapevine classification goal using NIR measurements acquired under field conditions with a portable device, while in [13] hyperspectral imaging was performed using a camera under laboratory conditions and having full control of illumination status.

The discrimination and classification of grapevine varieties using in-field NIR spectroscopy can be feasible as ated in this work. The variation in spectral properties in relation to leaf biochemical composition and structure, which depends on many factors like the plant species, the developmental or microclimate position of the leaf on the plant [37], have been outlined as potent factors causing this spectra differentiation. Still, the leaf water content can be an influence due to the fact that the absorption band of water can be found at 1940 nm. However, in this work the differences in water content have not driven the discrimination of leaves according to their variety, as special care was paid to measure only leaves with RWC marks between 80% and 90%.

In regard to spectra pretreatments (scatter correction and smoothing filtering), [38] remarked that selection of suitable spectral pre-treatment is not easy, due to the strong likelihood of several different mathematical transformation being used. The selection of the best pre-treatment for spectra analysis must be based on the combination of statistical testing and the modeller’s judgement [39]. Several factors can affect the results of applying different spectral pre-processing methods, ranging from sample nature to light conditions or spectra acquisition device’s status, etc, so the fact that SNV followed by de-trending and a second-degree derivative, window size 5 Savitzky-Golay filter cast the best classification marks would not assure this behaviour will maintain in other plant varietal discrimination problems.

In every case, SVM and ANN outperformed PLS-DA, whether five or 20 classes were used in the training. PLS-DA extracts the principal components from the whole set of wavelengths, by linear combinations of them, and ranking them depending on the more explained variance. The fact that SVMs and ANNs develop non-linear models and the complete set of wavelength values are used may be the cause of this better performance (e.g., the training process of an artificial neural network already penalizes those input variables less useful in the discrimination goal); this was more clear when comparing the N = 20 models. Although ANN performed better than SVM in almost every case, the lack of statistical significance between their scores allows to affirm that both of them could be used for this type of varietal discrimination purposes.

The outcomes the global method have thrown support the applicability of ANN for a multi-vineyard, vintage and phenological stage grapevine varietal discrimination. Despite the lower score obtained from the global vineyard dataset, 77.08%, versus the one achieved by the site-specific dataset, 87.25%, these results accomplished the classification goal with a high level of satisfaction, specially when taking into account that the model contemplated samples from different vineyards, seasons and leaf age, according to different phenological stages. Divergences were found regarding the pre-processing and parameter set combination with the best scores for site-specific and global datasets. While the algorithm (ANN) and smoothing filtering (D2W5) remained the same for both, not applying SNV and de-trending worked better in the global dataset, unlike the original one, where this scatter correction method gave a better response. This behavior could be a consequence of the differences in phenological stages and vineyards’ places at which the spectral measurements were acquired, given that leaf’s maturity is influenced by the time of year, and phenotype is affected by the physiology and field environment of the grapevine [40–42]. A scatter correction step might hide the spectral representation of these phenomena. Still, as previously discussed, the use of SNV and de-trending showed no statistical influence in the results, so the divergence related to the employment of this scatter correction procedure brought no big issue. The same applies for the ANN parameter set, where the original dataset responded better with a different one (parameter set 10) than the global dataset (parameter set 6). The minor particularities of the Multilayer Perceptron ANN implementation (such as the concrete values of the configuration parameters) are always very influenced by the input data and experimenter criteria, and could not be generalized.

The high marks obtained in the present work by both studied datasets, attending specially to the large number of classes for the site-specific model and the significant heterogeneity for the global one, opens several ways of direct application for viticulture and wine industry, including precision viticulture, if spectral data are georeferenced. In addition to the novelty of the spectral range and the high number of classes discriminated, it is worth highlighting that the spectra used in this study were acquired in the field, where illumination conditions are far from being stable. ANNs have demonstrated a notable accuracy for both potential applications of: a vineyard-specialized varietal classification, e.g., given vineyard plots sharing environmental, climatic and seasonal features (as evidenced by the 20-class model); and the global and generalized classification of vineyards from heterogeneous sources (different sites, vintages and phenological stages), such as those found in a whole region or territory. But this reliability is not the only valuable feature of this method: the easy usage because of its fast, portable and non-destructive nature makes the present grapevine varietal discrimination approach prone for direct in-field applicability by commercial vineyards, nurseries, appellation boards, among others. The remarkable performance of the developed model under field conditions paves the way for the use of this type of portable NIR analyses as powerful phenotyping tools in viticulture and other crops.

## Conclusions

The present study proposes a new classification method for the classification of grapevine varieties from in-field leaf NIR spectroscopy acquired through non-destructive methods. Modelling was approached in two ways: training the classifier with leaves from 20 varieties—building a site-specific model—and leaves from different vineyards, vintages and stages of development—building a global model. Support Vector Machines and Artificial Neural Networks showed a high reliability in the creation of grapevine leaf varietal classification models from in-field NIR spectroscopy using non-destructive data acquisition. The accuracy showed by both site-specific models, specially when the number of classes was high, along with the ability of properly train the model from heterogeneous sources, allows to consider this NIR range suitable for in-field grapevine varietal discrimination.

The classification results cast by the trained models open a new window in viticulture and wine industry, specially due to its portable and non-destructive nature, allowing the fast and in-field discrimination of a high number of grapevine varieties.

## References

[1] Galet P. A practical ampelography. Cornell University Press; 1979.

[2] Altube H, Cabello F, Ortiz JM. Caracterización de variedades y portainjertos de vid mediante isoenzimas de los sarmientos. Vitis. 1991;30(4):203–212.

[3] Sefc KM, Lefort F, Grando MS, Scott KD, Steinkellner H, Thomas MR. Microsatellite markers for grapevine: a state of the art. In: Molecular Biology & Biotechnology of the Grapevine; 2001 p. 433–463. 10.1007/978-94-017-2308-4_17

[4] Borrego J, De Andrés MT, Gómez JL, Ibáñez J. Genetic study of Malvasia and Torrontes groups through molecular markers. American Journal of Enology and Viticulture. 2002;53(2):125–130.

[5] Pelsy F, Hocquigny S, Moncada X, Barbeau G, Forget D, Hinrichsen P, et al An extensive study of the genetic diversity within seven French wine grape variety collections. Theoretical and Applied Genetics. 2010;120(6):1219–1231. 10.1007/s00122-009-1250-8 20062965

[6] Fernández-Novales J, López MI, Sánchez MT, García-Mesa JA, González-Caballero V. Assessment of quality parameters in grapes during ripening using a miniature fiber-optic near-infrared spectrometer. International Journal of Food Sciences and Nutrition. 2009;60(sup7):265–277. 10.1080/09637480903093116 19626519

[7] Pérez-Marín D, Paz P, Guerrero JE, Garrido-Varo A, Sánchez MT. Miniature handheld NIR sensor for the on-site non-destructive assessment of post-harvest quality and refrigerated storage behavior in plums. Journal of Food Engineering. 2010;99(3):294–302. 10.1016/j.jfoodeng.2010.03.002

[8] Wang W, Paliwal J. Spectral data compression and analyses techniques to discriminate wheat classes. Transactions of the ASABE. 2006;49(5):1607–1612. 10.13031/2013.22035

[9] Li X, He Y, Fang H. Non-destructive discrimination of Chinese bayberry varieties using Vis/NIR spectroscopy. Journal of Food Engineering. 2007;81(2):357–363. 10.1016/j.jfoodeng.2006.10.033

[10] Fu X, Zhou Y, Ying Y, Lu H, Xu H. Discrimination of pear varieties using three classification methods based on near-infrared spectroscopy. Transactions of the ASABE. 2007;50(4):1355–1361. 10.13031/2013.23613

[11] Xu HR, Yu P, Fu XP, Ying YB. On-site variety discrimination of tomato plant using visible-near infrared reflectance spectroscopy. Journal of Zhejiang University Science B. 2009;10(2):126–132. 10.1631/jzus.B0820200 19235271 PMC2644753

[12] Sánchez MT, De la Haba MJ, Benítez-López M, Fernández-Novales J, Garrido-Varo A, Pérez-Marín D. Non-destructive characterization and quality control of intact strawberries based on NIR spectral data. Journal of Food Engineering. 2012;110(1):102–108. 10.1016/j.jfoodeng.2011.12.003

[13] Diago MP, Fernandes AM, Millan B, Tardaguila J, Melo-Pinto P. Identification of grapevine varieties using leaf spectroscopy and partial least squares. Computers and Electronics in Agriculture. 2013;99:7–13. 10.1016/j.compag.2013.08.021

[14] Fernandes AM, Melo-Pinto P, Millan B, Tardaguila J, Diago MP. Automatic discrimination of grapevine (Vitis vinifera L.) clones using leaf hyperspectral imaging and partial least squares. The Journal of Agricultural Science. 2015;153(03):455–465. 10.1017/S0021859614000252

[15] Cortes C, Vapnik V. Support-vector networks. Machine Learning. 1995;20(3):273–297. 10.1023/A:1022627411411

[16] Xie C, Wang Q, He Y. Identification of Different Varieties of Sesame Oil Using Near-Infrared Hyperspectral Imaging and Chemometrics Algorithms. PLOS ONE. 2014;9(5):e98522 10.1371/journal.pone.0098522 24879306 PMC4039481

[17] Yang X, Hong H, You Z, Cheng F. Spectral and Image Integrated Analysis of Hyperspectral Data for Waxy Corn Seed Variety Classification. Sensors. 2015;15(7):15578–15594. 10.3390/s150715578 26140347 PMC4541845

[18] Kong W, Zhang C, Liu F, Nie P, He Y. Rice seed cultivar identification using near-infrared hyperspectral imaging and multivariate data analysis. Sensors. 2013;13(7):8916–8927. 10.3390/s130708916 23857260 PMC3758629

[19] McCulloch WS, Pitts W. A logical calculus of the ideas immanent in nervous activity. The Bulletin of Mathematical Biophysics. 1943;5(4):115–133. 10.1007/BF02478259 2185863

[20] Werbos P. Beyond regression: New tools for prediction and analysis in the behavioral sciences. PhD Thesis. 1974;.

[21] Rumelhart DE, Hinton GE, Williams RJ. Learning representations by back-propagating errors. Nature. 1986;323(6088):533–536. 10.1038/323533a0

[22] Li X, He Y. Discriminating varieties of tea plant based on Vis/NIR spectral characteristics and using artificial neural networks. Biosystems Engineering. 2008;99(3):313–321. 10.1016/j.biosystemseng.2007.11.007

[23] Yang CW, Chen S, Ouyang F, Yang IC, Tsai CY. A robust identification model for herbal medicine using near infrared spectroscopy and artificial neural network. Journal of Food and Drug Analysis. 2011;19(1).

[24] Barrs HD, Weatherley PE. A re-examination of the relative turgidity technique for estimating water deficits in leaves. Australian Journal of Biological Sciences. 1962;15(3):413–428.

[25] Barnes RJ, Dhanoa MS, Lister SJ. Standard Normal Variate Transformation and De-trending of Near-Infrared Diffuse Reflectance Spectra. Applied Spectroscopy. 1989;43(5):772–777. 10.1366/0003702894202201

[26] Dhanoa MS, Lister SJ, Barnes RJ. On the scales associated with near-infrared reflectance difference spectra. Applied Spectroscopy. 1995;49(6):765–772. 10.1366/0003702953964615

[27] Savitzky A, Golay MJE. Smoothing and differentiation of data by simplified least squares procedures. Analytical Chemistry. 1964;36(8):1627–1639. 10.1021/ac60214a047

[28] Platt J. Sequential minimal optimization: A fast algorithm for training support vector machines. Technical Report MSR-TR-98-14, Microsoft Research. 1998;.

[29] Hornik K, Stinchcombe M, White H. Multilayer feedforward networks are universal approximators. Neural Networks. 1989;2(5):359–366. 10.1016/0893-6080(89)90020-8

[30] Talens P, Mora L, Morsy N, Barbin DF, ElMasry G, Sun DW. Prediction of water and protein contents and quality classification of Spanish cooked ham using NIR hyperspectral imaging. Journal of Food Engineering. 2013;117(3):272–280. 10.1016/j.jfoodeng.2013.03.014

[31] Ivorra E, Girón J, Sánchez AJ, Verdú S, Barat JM, Grau R. Detection of expired vacuum-packed smoked salmon based on PLS-DA method using hyperspectral images. Journal of Food Engineering. 2013;117(3):342–349. 10.1016/j.jfoodeng.2013.02.022

[32] Canaza-Cayo AW, Cozzolino D, Alomar D, Quispe E. A feasibility study of the classification of Alpaca (Lama pacos) wool samples from different ages, sex and color by means of visible and near infrared reflectance spectroscopy. Computers and Electronics in Agriculture. 2012;88:141–147. 10.1016/j.compag.2012.07.013

[33] Vanloot P, Bertrand D, Pinatel C, Artaud J, Dupuy N. Artificial vision and chemometrics analyses of olive stones for varietal identification of five French cultivars. Computers and Electronics in Agriculture. 2014;102:98–105. 10.1016/j.compag.2014.01.009

[34] Stevens A, Ramirez-Lopez L. An introduction to the prospectr package; 2013. R package version 0.1.3.

[35] Borchers HW. pracma: Practical Numerical Math Functions; 2015. R package version 1.8.3.

[36] Hall M, Frank E, Holmes G, Pfahringer B, Reutemann P, Witten IH. The WEKA Data Mining Software: An Update. SIGKDD Explorations. 2009;11(1):10–18. 10.1145/1656274.1656278

[37] Jacquemoud S, Ustin SL. Leaf optical properties: A state of the art. In: 8th International Symposium of Physical Measurements & Signatures in Remote Sensing; 2001. p. 223–332.

[38] Fernández-Cabanás VM, Garrido-Varo A, Pérez-Marín D, Dardenne P. Evaluation of pretreatment strategies for near-infrared spectroscopy calibration development of unground and ground compound feedingstuffs. Applied Spectroscopy. 2006;60(1):17–23. 10.1366/000370206775382839 16454905

[39] Delwiche SR, Reeves JB. The effect of spectral pre-treatments on the partial least squares modelling of agricultural products. Journal of Near Infrared Spectroscopy. 2004;12:177–182. 10.1255/jnirs.424

[40] Sultan SE. Phenotypic plasticity for plant development, function and life history. Trends in Plant Science. 2000;5(12):537–542. 10.1016/S1360-1385(00)01797-0 11120476

[41] Nicotra AB, Atkin OK, Bonser SP, Davidson AM, Finnegan EJ, Mathesius U, et al Plant phenotypic plasticity in a changing climate. Trends in Plant Science. 2010;15(12):684–692. 10.1016/j.tplants.2010.09.008 20970368

[42] Pélabon, Osler NC, Diekmann M, Graae BJ. Decoupled phenotypic variation between floral and vegetative traits: distinguishing between developmental and environmental correlations. Annals of botany. 2013;111(5):935–944. 10.1093/aob/mct050 23471008 PMC3631334

